# Extracellular Vesicles as Next-Generation Diagnostics and Advanced Therapy Medicinal Products

**DOI:** 10.3390/ijms25126533

**Published:** 2024-06-13

**Authors:** Agnieszka Stawarska, Magdalena Bamburowicz-Klimkowska, Elise Runden-Pran, Maria Dusinska, Mihaela Roxana Cimpan, Ivan Rios-Mondragon, Ireneusz P. Grudzinski

**Affiliations:** 1 Department of Toxicology and Food Science, Faculty of Pharmacy, Medical University of Warsaw, Banacha Str. 1, 02-097 Warsaw, Poland; magdalena.bamburowicz-klimkowska@wum.edu.pl (M.B.-K.); ireneusz.grudzinski@wum.edu.pl (I.P.G.); 2 Health Effects Laboratory, Department of Environmental Chemistry, Norwegian Institute for Air Research, 2007 Kjeller, Norway; erp@nilu.no (E.R.-P.); mdu@nilu.no (M.D.); 3 Biomaterials—Department of Clinical Dentistry, Faculty of Medicine, University of Bergen, Årstadveien Str. 19, 5009 Bergen, Norway; mihaela.cimpan@uib.no (M.R.C.); ivan.rios-mondragon@uib.no (I.R.-M.)

**Keywords:** extracellular vesicles, diagnostics, medicinal products, GMP manufacturing, preclinical studies, clinical trials, regulatory affairs

## Abstract

Extracellular vesicles (EVs) hold great promise for clinical application as new diagnostic and therapeutic modalities. This paper describes major GMP-based upstream and downstream manufacturing processes for EV large-scale production, also focusing on post-processing technologies such as surface bioengineering and uploading studies to yield novel EV-based diagnostics and advanced therapy medicinal products. This paper also focuses on the quality, safety, and efficacy issues of the bioengineered EV drug candidates before first-in-human studies. Because clinical trials involving extracellular vesicles are on the global rise, this paper encompasses different clinical studies registered on clinical-trial register platforms, with varying levels of advancement, highlighting the growing interest in EV-related clinical programs. Navigating the regulatory affairs of EVs poses real challenges, and obtaining marketing authorization for EV-based medicines remains complex due to the lack of specific regulatory guidelines for such novel products. This paper discusses the state-of-the-art regulatory knowledge to date on EV-based diagnostics and medicinal products, highlighting further research and global regulatory needs for the safe and reliable implementation of bioengineered EVs as diagnostic and therapeutic tools in clinical settings. Post-marketing pharmacovigilance for EV-based medicinal products is also presented, mainly addressing such topics as risk assessment and risk management.

## 1. Introduction

**Figure 1:**
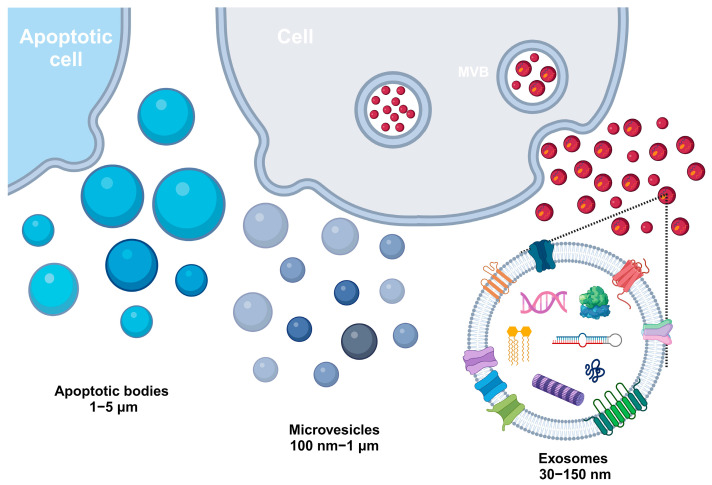
Classification of extracellular vesicles—three types of EVs: apoptotic bodies, microvesicles, and exosomes. Abbreviations: EVs—extracellular vesicles; MVB—multivesicular bodies.

By the term “extracellular vesicles” (EVs), according to the currently accepted nomenclature, we mean heterogeneous vesicles of cellular origin, surrounded by a lipid bilayer, incapable of self-replication (not containing a functional nucleus), and which are produced by most cells through various mechanisms [1]. So far, EVs have been detected in plants [2,3,4], bacteria [5,6,7,8], fungi [6,9], in vitro cultures of eukaryotic cells, and biological samples obtained from humans and animals [10,11,12,13]. Established in 2011, the International Society of Extracellular Vesicles (ISEV) has updated the EV nomenclature. Currently, three types of EVs are distinguished, namely (i) microvesicles (MVs) or ectosomes, (ii) apoptotic bodies, and (iii) exosomes (Figure 1) [14]. MVs refer to EVs in the range of 100–1000 nm that are formed at and directly secreted from the cell membrane through a complex mechanism consisting of exfoliation or budding under normal circumstances or in response to different stimuli [15]. Several proteins have been identified as specific biomarkers of MVs, including CD40, ribosylation factor ADP 6 (ARF6), selectin, phosphatidylserine, and members of the Rho family of GTPases [16]. Apoptotic bodies (1000–5000 nm) are membrane-bound cellular remains released when programmed cell death is induced and contain DNA fragments that do not encode RNA and cellular organelles [17]. Annexin V and histones are reported as specific proteins of apoptotic bodies [18,19,20]. Exosomes are EVs with a size range of 30–150 nm, which are heterogeneous in both size and cargo composition. Therefore, exosomes have diverse physiological roles depending on the donor and target cells [21]. Exosomes have been reported to contain several types of specific surface markers, such as tetraspanins (CD9, CD63, and CD81), heat shock proteins (Hsp70 and Hsp90), MVB synthesis proteins, and membrane transporters and fusion proteins (annexins and flotillins) [22,23]. Although the origins of each type of vesicle and the corresponding markers have been determined, available technologies are not able to provide pure isolates of each type of EV since there is some overlap of physicochemical properties and molecular markers. This is why the ISEV, in its latest position paper issued in 2023 (MISEV2023), recommends the use of the generic term “EVs” instead of inconsistently mentioned and sometimes misleading terms, such as “exosomes” and “ectosomes”, which are associated with difficult-to-determine biogenesis pathways [1].

During the last decade, huge interest has arisen in the studies of EV subclasses, referred to here as exosomes, that are secreted by various cell types and are composed of lipids, proteins, and nucleic acid cargos [24]. Note that exosomes are formed through the inward budding of the endosomal membrane, resulting in the formation of intraluminal vesicles (ILVs) within multivesicular bodies (MVBs). Both endosomal sorting complex required for transport (ESCRT) and non-ESCRT systems are involved in these processes [25]. Interestingly, MVBs can then fuse with the plasma membrane, releasing the ILVs into the extracellular environment, and they are further referred to as exosomes [25]. After being released, EVs affect recipient cells at the molecular level through several mechanisms, which were described in details by Krylova at al [26]. This may include soluble signaling, which involves the proteolytic cleavage of ligands from the exosome surface, or alternative splicing, whereas juxtacrine signaling requires the assembly of ligands and receptors on the exosome and target cell surface. EVs can interact with surface receptors on recipient cells, triggering signal transduction pathways. This interaction can lead to changes in cellular behavior, such as proliferation, differentiation, or apoptosis. Then, EVs can fuse with the plasma membrane of recipient cells, directly delivering their cargo into the cytoplasm. This allows for the rapid and efficient transfer of functional molecules. Moreover, recipient cells can internalize EVs through endocytosis. Once inside, the EVs can be processed in endosomes and lysosomes, releasing their contents into the cytoplasm, affecting intracellular signaling pathways.

**Figure 2:**
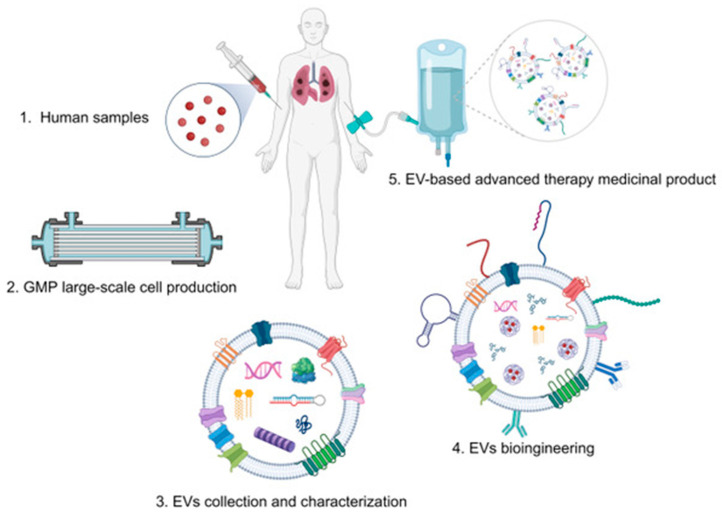
Extracellular vesicles (EVs) as personalized biological drug products. Abbreviations: GMP—Good Manufacturing Practice.

Exosomes play important roles in cell-to-cell communication and are thought to function in a variety of physiological and pathological processes, including immune modulation, tissue regeneration, and cancer progression [27]. Exosomes can transfer a variety of molecular cargos, including proteins, lipids, and nucleic acids, altering the phenotype and behavior of recipient cells [28]. The protein content of exosomes is highly diverse, and it can vary depending on the cell type that secretes them [29,30,31,32]. Lipids in exosomes include phospholipids, cholesterol, and sphingolipids [33]. The nucleic acids in exosomes are primarily composed of RNA species, including messenger RNA, microRNA, and other small non-coding RNA species [33]. Exosomes can cross biological barriers, such as the blood–brain barrier, and have the ability to deliver their cargo to specific target cells [28,33,34]. Due to their ability to transfer biologically active molecules, exosomes have garnered significant interest in recent years as potential therapeutic agents or diagnostic tools [35,36] (Figure 2).

Since EVs circulate in almost all types of bodily fluids, such as blood, urine, saliva, broncho-alveolar fluid, breast milk, and semen, the collection of which is of a non-invasive nature, they also have huge potential as biomarkers of many diseases. These include tumors [22,37,38,39], bone-related diseases [13], amyotrophic lateral sclerosis [40], traumatic brain injury [41], lung disease [42], liver disease [43,44], inflammatory eyes disease [45], helminthic infection [46], autoimmune diseases [47], and kidney disease [48]. It has been shown that the analysis of the composition of EVs (proteins, miRNA, mRNA, and lipids) can be useful not only in diagnosing but also in assessing the progression of the disease [22,49,50,51,52,53,54,55]. Depending on their origin, the EVs can modulate the morphology and metabolism of the target cells, which can lead to pathogenesis. EVs, notably exosomes, serve as distinctive messengers facilitating communication between tumors and host organisms. They are extensively investigated as endogenous nanoscale transporters carrying various molecules in both physiological and pathological conditions. Research indicates that EVs play a pivotal role in processes such as thrombosis, angiogenesis, vascular dysfunction, and others, influencing the progression of various hematologic diseases, including cancers [56]. For example, in hematological malignancies, EVs originating from cancer cells assume a critical role in facilitating intercellular communication. They accomplish this by transferring genetic materials, proteins, and other as-yet-unidentified molecules between cancer cells and the bone-marrow microenvironment. This transfer plausibly influences processes such as cell transformation, proliferation, and ultimately the progression of malignancy [57]. Due to the ability of EVs to deliver bioactive molecules and cross biological barriers, EVs are increasingly being considered as potential therapeutic agents [22,58,59,60,61,62] (Figure 2).

## 2. Extracellular Vesicles Loaded with Diagnostic and Therapeutic Cargos

**Figure 3:**
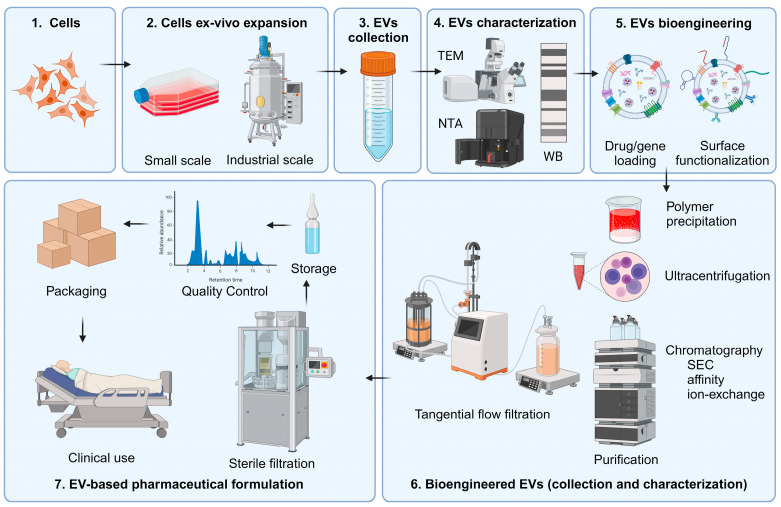
Overall EVs’ GMP-based manufacturing workflow. Abbreviations: EVs—extracellular vesicles; SEC—Size-Exclusion Chromatography; GMP—Good Manufacturing Practice; TEM—Transmission Electron Microscopy; NTA—Nanoparticle Tracking Analysis; WB—Western Blot.

The use of extracellular vesicles (EVs) as new biological transporters for different diagnostic or therapeutic moieties requires the large-scale production of cellular mass with Good Manufacturing Practice (GMP) regulations [63]. In most of the recent studies, the source of EVs was mesenchymal stromal cells, adipose tissue-derived stem cells, and human embryonic kidney cells [64]. However, the EV samples could also be collected from some bacterial cultures, plant cells, and even bovine milk [65]. Although obtaining EVs from human fluids, such as blood and plasma, does not require specific cell-culture technologies, the large-scale production of EVs from a specific cellular mass will need standardized cell-culture approaches following GMP protocols [64] (Figure 3).

Manufacturing EVs includes the so-called upstream and downstream processes [66]. The upstream processes are related to cell banking and cell expansion for the production of conditioned media [67,68]. It should be emphasized that setups based on cell culture in conventional 2D flask systems are limited to the production of small number of EVs, and this is a problem for commercial purposes. Therefore, 3D cell culture systems have been developed. These include stirred-tank, vertical wheel, rocker, and hollow-fiber bioreactors [69]. Notably, the hollow-fiber bioreactor is widely used for EV production. This system is composed of thousands of parallel hollow fibers whose membrane supports cell attachment and the transport of small molecules (nutrients) from the fiber lumen to the extra-capillary space where the cell mass resides [70]. Furthermore, flow control systems continuously deliver fresh or recirculated cell culture medium to the lumen of the fibers to support cell growth, while the harvesting of EV-conditioned medium from the extra-capillary space is performed at specific time intervals. This unique construction and operation allow for the production of a large cell mass and high number of EVs [71]. The optimal production of a large cell mass in hollow-fiber bioreactors should take into account the quality control of parameters such as the pH; temperature; osmolarity; hypoxia; and nutrients added to the medium, including glucose and ion contents [72]. Therefore, the upscaling of hollow-fiber bioreactors from preclinical studies to manufacturing scale requires numerous iterative tests to keep the cell mass phenotype acceptable while producing EV batches on a large scale (Supplementary Figure S1). Evidence from recent studies indicates that the large-cell mass production in hollow-fiber bioreactors could be applied for the manufacturing of EVs following GMP standards [73].

Creating therapeutic EVs involves several key steps, each critical to ensure the quality and efficacy of the final biological product. Quality control (QC) measures and standards in EV production encompass rigorous assessment of source cells, isolation and purification methods, EV characterization, functional assays, stability testing, and regulatory compliance. By implementing these QC measures, manufacturers can ensure consistency, safety, and efficacy of EV-based products for biomedical applications. The ongoing monitoring and continuous improvement of QC processes are essential to maintain product quality and meet regulatory standards. A detailed diagram of the EVs’ production, including the purpose, process description, and quality control at each stage, is presented in Table 1 and Figure 3.

Scaling up EV production for clinical and commercial use presents challenges in consistency, efficiency, and cost-effectiveness. Optimizing culture systems, such as bioreactors, and engineering source cells can enhance proliferation and EV secretion. Improved isolation methods, like tangential flow filtration and automation, can increase throughput and purity. Standardized protocols and real-time monitoring ensure quality and safety. Cost-saving measures, novel technologies, and regulatory compliance are essential for successful upscaling. Technology transfer plans and personnel training facilitate seamless production. Collaboration is key to advancing EV-based therapies and diagnostics efficiently and safely.

There are different downstream processes used for the large-scale production of EVs, such as (i) differential ultracentrifugation with density-gradient centrifugation, (ii) filtration paired with centrifugation and immunoaffinity capture, (iii) Size-Exclusion Chromatography (SEC), (iv) polymer-based precipitation, and (v) microfluidics technologies [33,36,74]. All of these methods have their own advantages and disadvantages (Table 2 and Figure 3). Nowadays, differential ultracentrifugation-based techniques are the gold standard for EV isolation. In this case, EV isolation is based on density and size, with the possibility of sequential separations of large volumes of samples. However, this technique is time-consuming; it requires large starting volumes, characterized by high susceptibility to operator-based variability; and exosomes may be damaged by high-speed centrifugation. Filtration—in particular, ultrafiltration—for EV isolation is an operational, simple, high-throughput, cost- and time-effective separation process. Unlike batch-wise ultracentrifugation, ultrafiltration can be operated in continuous mode, which makes this downstream separation technique suitable for large-scale production. However, potential EV degradation and lysis due to the shear forces and the clogging and trapping of exosomes on to the filter membrane (and therefore exosome loss) are risks that should be considered when applying filtration. High-purity EVs with no disturbances in their structure, integrity, and biological activity are obtained using SEC. This method offers high reproducibility and can be operated in pseudo-continuous mode with a high degree of automation. Microfluidics-based techniques and immune-affinity capture methods based on affinity, size, and density principles give highly specific and pure fraction of exosomes with fast sample processing, high efficiency, and high portability. Most methods have their usefulness depending on the application, but it should be remembered that each of the methods to a greater or lesser extent may not be suitable for large-scale EV purification [74]. After isolation, EVs should be thoroughly characterized by multiple methods to validate the isolation method. For exosomes, the most common characterization methods can be classified as marker-based transmembrane proteins (CD63, CD9, and CD81) or intraluminal proteins (TSG101 and ALIX), biophysical (dynamic light scattering, flow cytometry, nanoparticle tracking analysis, and tunable resistance pulse sensing), or imaging-based (atomic force microscopy and electron microscopy) [36]. EV characterization also includes cargo profiling by characterizing proteins, nucleic acids, lipids, and metabolites, using omics approaches such as proteomics, genomics, and lipidomics to ensure cargo consistency.

Due to the characteristic features of EVs, including low immunogenicity, innate stability, and the ability to cross biological barriers, the usefulness of EVs as therapeutic carriers to deliver various types of cargo molecules to the target cancer cells began to be investigated [75,76,77]. Comparing exosomes to synthetic liposomes having a similar structure of the lipid bilayer, we see that bio-derived exosomes are characterized by an increased ability to load biological molecules and greater efficiency in reaching target cells and delivering therapeutic agents when administered intravenously [78]. When using EVs to deliver active molecules, many approaches have been explored to load EVs with various drugs and other bioactive compounds. In general, there are two different approaches to load cargos into EVs, defined as (i) pre-loading and (ii) post-loading methods [33]. In pre-loading, the parental cells are engineered to produce bioactive molecules that are encapsulated into EVs by the natural sorting process during EV maturation. Thus, EV-producing cells secrete EVs loaded with the bioactive molecule of interest. In post-loading, drugs and bioactive molecules are directly loaded into EVs after EV isolation.

The gold standard for the uploading of exogenous materials into EVs is electroporation, where an electric pulse temporally generates pores in the lipid bilayer, and exogenous cargo can diffuse into the inner space of EVs. Recently, numerous nanomaterials, drugs, and nucleic acids have been electroporated into EVs. Pan et al. (2020) used exosomes isolated from the urine of gastric cancer patients for tumor diagnosis and therapy [79]. In this study, gold nanoparticles coated with polymethacrylates, in conjunction with chlorin-loaded exosomes, were used for cancer imaging and photodynamic therapy [79]. Chemotherapeutic drugs, such as paclitaxel and doxorubicin, have been loaded via electroporation into macrophage-derived EVs [80] and immature dendritic cell-derived EVs [81], respectively. Electroporation is also utilized to directly load nucleic acids into exosomes. Alvarez-Erviti et al. (2011) obtained siRNA-loaded exosomes, which possessed the ability to knock down β-secretase 1 in mouse brains [82], while in murine dendritic cell-derived exosomes loaded with shRNA, they allowed for a decrease in α-synuclein aggregation and alleviated dopaminergic neuron loss in PD models [83]. Sonication is a physical method that uses ultrasounds to weaken the integrity of the EV membrane, which facilitates the loading of exosomal cargo, including drugs [84], proteins [85], and nanomaterials [86]. Extrusion is a physical procedure that involves compressing a mixture of EVs, especially sized 30–150 nm, and cargo in an extruder to induce the recombination of the EV membrane so that the membrane collapses and uniformly mixes with the cargo [36]. Fuhrmann et al. [87] extruded the exosomal samples to load porphyrins. Haney et al. [85] used this strategy and limited cycles of rapid freezing and thawing to produce catalase-loaded exosomes. However, repeated freeze–thaw could inactivate proteins and induce exosome aggregation [34]. The abovementioned physical methods allow EVs to be loaded with a higher efficiency than simple cargo and EVs’ co-incubation. Nevertheless, recent reports demonstrate that many types of cargoes can be successfully loaded into EVs using this simple method. Paclitaxel and doxorubicin have shown enhanced chemotherapeutic effects after being loaded into prostate cancer cell-derived [88] and mesenchymal stromal/stem cell-derived exosomes [89]. Gong et al. applied this strategy to nucleic acids packaging and produced miRNA-loaded macrophage-derived exosomes [89,90]. Gold nanoparticles-labeled exosomes for noninvasive in vivo neuroimaging and tracking were obtained by Batzer et al. [91]. Encapsulation of withaferin A, anthocyanidins, curcumin, paclitaxel, docetaxel, and catalase into EVs has been achieved by incubating those compounds with EVs in the presence of organic solvents, such as ethanol, or detergents, such as saponin, to create pores on the exosomal surface, thus leading to an increase in membrane permeability [85,88,92].

EVs arming can also be achieved via genetic engineering, which involves the modification of the exosome-producing cells to produce exosomes with desired properties or cargoes. One common approach is to modify the EV-producing cells to overexpress a specific protein or peptide of interest, which can then be selectively packaged into the exosomes. This can be achieved by introducing a plasmid or viral vector containing the gene of interest into the cells and selecting for cells that stably express the gene [33,74,93]. Alternatively, CRISPR-Cas9 technology can be used to knock out or knock in specific genes to manipulate the cargo of the exosomes [94,95]. Another approach is to modify the exosome-producing cells to express a chimeric protein that contains a targeting moiety, such as an antibody or peptide, fused to a protein that directs the protein to the exosome membrane [36]. This can result in the specific packaging of the targeting moiety into the exosomes and selective targeting of specific cells or tissues. In addition to cargo modification, genetic engineering can also be used to modify the properties of the exosomes themselves, such as their size, shape, or surface charge. For example, the exosome-producing cells can be modified to overexpress specific membrane proteins or lipids that can affect the properties of the exosomes [34]. Genetic engineering of exosomes can provide a powerful approach for functionalizing exosomes with specific cargoes or properties and for achieving selective targeting of specific cells or tissues. However, it is important to note that genetic modification can affect the safety and efficacy of the exosomes and may require careful evaluation and regulation before their use in clinical applications [28,96].

## 3. Surface Bioengineered Extracellular Vesicles as Targeted Delivery Systems

To achieve targeted delivery, the surface of EVs can be modified by functionalization with specific ligands or antibodies that bind to specific receptors on the target cells. Here are some common methods for exosome surface functionalization. A basic method is covalent conjugation, which involves attaching specific ligands or antibodies to the surface of exosomes by forming covalent bonds between functional groups on the exosome surface and the ligand or antibody [33,97]. Then comes the non-covalent conjugation, which involves adsorption of the ligands or antibodies onto the surface of exosomes through non-covalent interactions, such as electrostatic interactions or hydrophobic interactions [33,97]. Another method involves lipid membrane modifications resulting in incorporating modified lipids with specific ligands or antibodies into the exosome membrane, which can then be recognized by target cells [36,98]. More recently, genetic engineering was used where genetically modified cells were enabled to produce EVs that express specific ligands or antibodies on the surface of EVs [33,98]. The choice of method for EV surface functionalization depends on the specific application and the properties of the ligands or antibodies being used. Successful functionalization can improve the specificity and efficiency of exosome-based therapeutics, making them promising tools for targeted drug delivery and personalized medicine [33]. Covalent conjugation is a commonly used method to achieve this because it involves the formation of a stable chemical bond between the surface of the exosome and the molecule of interest. The molecule of interest is usually modified with a functional group such as a thiol (-SH), amine (-NH2), or carboxyl (-COOH) group that can react with a complementary functional group on the exosome surface [99]. One common approach for covalent conjugation is to use maleimide chemistry. Maleimide groups react specifically with thiols to form a stable thioether bond. Thus, molecules with thiol groups, such as cysteine residues in proteins, can be conjugated to maleimide-functionalized exosomes [100]. Another approach is to use azide–alkyne cycloaddition or click chemistry, which produces no alterations in exosome size and function [28,98]. Covalent conjugation can be used to target exosomes to specific cells or tissues by attaching targeting moieties such as antibodies or peptides that recognize cell surface receptors [33,97]. Additionally, it can be used to deliver therapeutic molecules such as small interfering RNA (siRNA) or chemotherapeutic drugs to specific cells or tissues [82,101]. However, it is important to note that covalent conjugation can affect the biological properties of exosomes and may alter their biodistribution and clearance in vivo [98]. Therefore, careful characterization and evaluation of the functionalized exosomes is essential before their use in biomedical applications.

Non-covalent conjugation is an alternative method for functionalizing EVs that does not involve the formation of a stable chemical bond between the surface of the exosome and the molecule of interest. Instead, non-covalent conjugation relies on weak, reversible interactions such as electrostatic interactions, hydrophobic interactions, or hydrogen bonding [28,102]. One common approach for non-covalent conjugation is to use the liposome fusion. Note that liposomes are artificial vesicles made of phospholipids that can fuse with the exosome membrane and deliver their cargo into the exosome lumen or surface. The liposomes can be functionalized with various molecules, such as proteins, peptides, or nucleic acids, which can then be transferred to the exosome surface or lumen [97,98]. Another approach is to use a biotin–streptavidin conjugation. Biotinylated molecules can bind to streptavidin-coated exosomes via biotin–streptavidin interactions, which are one of the strongest non-covalent interactions in nature. Streptavidin can also be conjugated to various molecules, such as antibodies, peptides, or nucleic acids, which can then be delivered to the exosome surface or lumen [33,103]. Non-covalent conjugation has several advantages over covalent conjugation. It is a gentle method that does not require harsh chemical reactions, which can affect the biological properties of exosomes. It also allows for reversible binding, which can facilitate the release of the cargo at the target site. However, non-covalent conjugation can also have lower efficiency and specificity than covalent conjugation, and the cargo may dissociate from the exosome under certain conditions. In summary, both covalent and non-covalent conjugation are valuable methods for functionalizing exosomes, and the choice of method depends on the specific application and the properties of the cargo and the exosome [98,104].

EVs subclassed as exosomes are surrounded by a lipid bilayer membrane that contains various lipids and proteins, and modifications of this membrane can be used to functionalize exosomes. One approach for membrane modification is to insert lipids with functional groups or ligands into the exosome membrane. One common type of modified lipid is polyethylene glycol (PEG)-modified lipids, which can improve the stability and circulation time of exosomes in vivo by reducing their clearance by the immune system [76,97]. Another approach is to insert lipids with targeting moieties or ligands, such as antibodies or peptides, into the exosome membrane to achieve selective targeting of specific cells or tissues. This can be achieved by coupling the targeting moiety to a lipid molecule that can be incorporated into the exosome membrane, such as phosphatidylcholine or cholesterol. The targeting moiety can then bind to specific receptors on the target cells or tissues. In addition to lipid modification, the exosome membrane can also be modified by attaching proteins or peptides to the surface of the exosomes. This can be achieved by fusing the protein or peptide to a lipid anchor that can insert into the exosome membrane [33,34,74]. The modification of the exosome membrane can provide a versatile approach for functionalizing exosomes with various molecules and achieving selective targeting of specific cells or tissues. However, it is important to note that membrane modification can affect the biological properties of exosomes and may alter their interactions with cells and tissues. Therefore, careful characterization and evaluation of the modified exosomes is essential before their use in biomedical applications [74,95,98].

Functionalized EVs have shown great potential for both cancer treatment and diagnosis. Recent studied evidenced that functionalized exosomes can be used to deliver therapeutic agents, such as small-molecule drugs [89,105,106], nucleic acids [82,83,101,107,108], or proteins [85], to cancer cells. EVs can also carry a mixture of different components, such as human vascular endothelial growth factor A (VEGF-A) and human bone morphogenetic protein 2 (*BMP-2*) mRNAs within a customized injectable PEGylated poly (glycerol sebacate) acrylate (PEGS-A) hydrogel for bone tissue regeneration [109]. In addition to their abovementioned uses, cargo EVs can be engineered to express IL6 signal transducer, which has the ability to inhibit the IL6 trans-signaling pathway and thus have anti-inflammatory effects [110]. By modifying the exosome surface with targeting moieties, such as antibodies or peptides, the exosomes can selectively bind to cancer cells and deliver their cargo, resulting in enhanced efficacy and reduced toxicity. Functionalized exosomes can also be used for immunotherapy by delivering antigens or immune modulators to stimulate the immune system against cancer cells (Figure 2) [111]. Note that exosomes can be isolated from various body fluids, including blood, urine, and cerebrospinal fluid, and their cargo can be analyzed to identify biomarkers for cancer diagnosis and monitoring. By modifying the exosome surface with targeting moieties, such as aptamers or antibodies, exosomes can be selectively captured and analyzed to detect cancer-specific biomarkers, such as proteins or nucleic acids [112,113]. Functionalized exosomes can also be used for imaging by loading them with contrast agents or fluorescent dyes to visualize cancer cells and tumors [35,91]. Studies evidence that functionalized exosomes can be used in combination with other cancer therapies, such as chemotherapy or radiation therapy, to enhance their efficacy and reduce their side effects [34,74,84,97,114]. By delivering therapeutic agents to cancer cells and stimulating the immune system, functionalized exosomes can enhance the tumor-killing effect of other cancer therapies and improve patient outcomes.

Due to the possibility of their functionalization, exosomes are increasingly used for targeting for various therapeutic purposes, and thus various possibilities of their applications in cancer therapy and diagnostics are being investigated [24,33,115]. Among other things, EVs are suitable candidates for improving the targeting of chemotherapeutic drugs. At present, chemotherapeutic-loaded EVs are described as internalizing in tumor cells to induce cell death [116], improving the cytotoxic effect of paclitaxel on LNCaP and PC3 cells [88], inhibiting and improving malignant U-87 cells growth in a dose-dependent manner [117], enhancing antiproliferative and anti-inflammatory activity in CFPAC-1 cells [116], and highly efficient targeting to αv-integrin-positive breast cancer cells in vitro and high specific delivery to tumor tissue without overt toxicity in a MDA-MB-231 cancer mouse model [81]. Moreover, nucleic acid-loaded EVs allowed for a reduction of cell proliferation and invasion and an increase in apoptosis and cell-cycle arrest, along with inhibited growth, in xenograft tumors in vivo [101]; the delivery of siRNA molecules and induction/downregulation of *TPD52* gene expression in SKBR3 breast cancer cells [107]; and decreased rates of cell migration and proliferation due to exosomes releasing miR26a from selectively bound HepG2 cells [118].

## 4. Clinical Trials on Extracellular Vesicles

EVs have been used, among other things, in the treatment of cancer [22,62,119,120,121], neurodegenerative diseases [122,123,124], cardiovascular disease [125,126,127], kidney disease [48], lung disease [42], and in regenerative medicine [128]. There are two different uses of EVs. In the first case, the specific biological properties of EVs, including their role in intercellular communication, are used to target a given tissue and reduce pathological signals or mimic the natural repair process. These properties are associated with a large number of proteins and lipids on the surface of the EVs. In the second case, EVs serve as carriers for delivering therapeutic agents to their destinations [129,130]. In recent years, the term “hybrid EV system” or “biohybrid” has been introduced to refer to the formula of nanoscale drug delivery systems consisting of (synthetic) conventional components and EVs. The use of biohybrid systems improves the arrival at the destination and the effectiveness of loading the drug [131].

**Figure 4:**
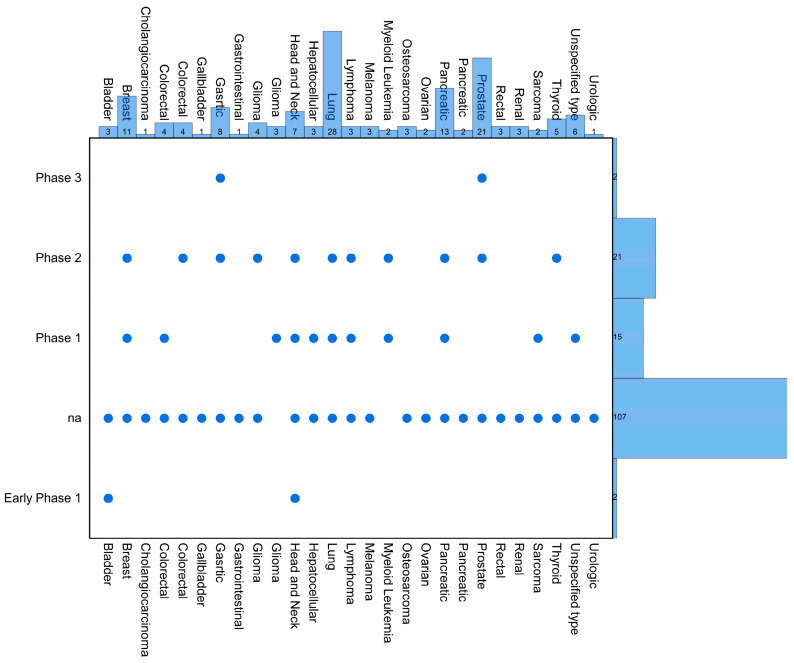
Clinical trials using exosomes in the treatment and diagnosis of cancer in the clinicaltrials.gov database.

Clinical studies on EVs subclassed as exosomes in cancer diagnostics have attracted significant attention due to the potential for the early detection and precise identification of various types of cancers. The research focuses on utilizing exosomes to identify cancer-specific biomarkers, providing valuable information for accurate diagnosis. Notable findings include the identification of cancer-specific biomarkers through the isolation and analysis of exosomes from cancer patients [132,133]. Exosomes show promise in regard to enabling the early detection of tumors and facilitating timely intervention and improved prognosis [134]. The potential for differentiating between various types of cancers using exosomal biomarkers is a key area of investigation, aiming to establish panels of biomarkers for improved diagnostic accuracy [135]. Additionally, investigations into the prognostic value of exosomal biomarkers connect specific exosome profiles to disease progression and prognosis, guiding treatment strategies and informing patients about the likely course of their cancer [136,137,138,139]. Research efforts have expanded to explore the clinical utility of exosomal biomarkers across various cancer types, enhancing the applicability of exosome-based diagnostics [136,140]. In the clinical trials.gov database, the most registered research concerns the diagnostic use of exosomes, which is illustrated in Figure 4. Several examples of research at various levels of advancement are provided: terminated trials—NCT04960956 Glycosylation of Exosomes in Prostate and Urothelial Carcinoma and NCT04357717 ExoDx Prostate Evaluation in Prior Negative Prostate Biopsy Setting; completed ones—NCT04394572 Identification of New Diagnostic Protein Markers for Colorectal Cancer and NCT05101655 Construction of Microfluidic Exosome Chip for Diagnosis of Lung Metastasis of Osteosarcoma; and with active recruiting—NCT05854030 A Study on Serum Exosomal miRNA Predicting the Effective and Prognosis of Immunotherapy Combined With Chemotherapy in Pulmonary Squamous Carcinoma.

In the context of cancer gene therapy, clinical studies on exosomes have emerged as a promising path for delivering genetic materials to target cells. Exosomes, with their unique properties, serve as valuable carriers for genetic materials, potentially modulating gene expression and improving therapeutic outcomes. Notable findings include the exploration of exosomes as carriers for delivering genetic materials, protecting and transporting therapeutic cargo to specific target cells [141,142,143]. Clinical research focuses on their potential in delivering RNA-based therapies, such as small interfering RNA (siRNA) or microRNA, for targeted gene silencing in cancer cells [144], with an example from the clinicaltrial.gov base—NCT03608631 Exosomes in Treating Participants With Metastatic Pancreas Cancer With KrasG12D Mutation. Investigations have also explored exosomes as carriers for CRISPR/Cas9 gene-editing tools, allowing for precise genome modifications [145,146]. The potential to enhance therapeutic efficacy by improving the stability and targeted delivery of genetic materials using exosomes is an area of active exploration. Ongoing clinical research is dedicated to translating exosome-based gene therapies from preclinical studies to human trials, evaluating safety and efficacy in cancer patients [147].

In the area of cancer drug delivery, clinical research on exosomes has become a promising field, exploring their potential to transport therapeutic agents to specific cells or tissues. Exosomes, being natural carriers released by cells, offer unique advantages in drug delivery. Research investigates modifying exosomes to carry targeting ligands or antibodies, minimizing side effects, and enhancing the specificity of drug delivery [95,98]. Studies have explored the use of exosomes as carriers for delivering anticancer drugs, protecting therapeutic payloads from degradation, and enhancing their targeted delivery to cancer cells [95]. Clinical research has also entered into modifying exosomes to carry specific targeting ligands or antibodies, allowing for the precise delivery of therapeutic drugs to cancer cells, while minimizing off-target effects [148]. The use of exosomes to deliver drugs with the aim of minimizing side effects is a focus of ongoing research, aiming to enhance the therapeutic index of anticancer medications [95]. Clinical studies have explored loading exosomes with multiple therapeutic agents for co-delivery, aiming to enhance treatment efficacy through synergistic effects [149]. Investigations have also explored exosomes’ potential to overcome multidrug resistance in cancer cells, encapsulating drugs and delivering them to resistant cells [148,150]. The significant promise of clinical research on exosomes in cancer drug delivery lies in the potential to develop more targeted and effective cancer treatments, minimizing side effects, and improving overall therapeutic outcomes, what the research NCT04453046 Hemopurifier Plus Pembrolizumab in Head and Neck Cancer and NCT01294072Study Investigating the Ability of Plant Exosomes to Deliver Curcumin to Normal and Colon Cancer Tissue may prove in the future.

In cancer neurology applications, clinical research on exosomes brings to light the complex interplay between exosomes and the nervous system in the context of cancer-associated neurological conditions. Understanding these mechanisms may open opportunities for diagnostic biomarkers and innovative therapeutic approaches for neurological diseases linked to cancer. Research in this area highlights the active participation of exosomes in mediating communication within the nervous system, playing a role in the transfer of various signaling molecules between different cell types [151]. Studies have focused on understanding the role of exosomes in neurological diseases associated with cancer, such as Alzheimer’s disease and multiple sclerosis [152,153,154]. Investigations explore the potential of exosomal biomarkers in identifying and understanding neurological conditions associated with cancer, offering specific molecular cargo as diagnostic or prognostic indicators [136,152,155]. Clinical research has also explored the therapeutic potential of exosomes in treating neurological disorders associated with cancer, considering the engineering of exosomes to deliver therapeutic agents to affected areas of the nervous system [156]. The role of exosomes in allowing communication between cancer cells in the brain microenvironment during metastasis is also under investigation [157,158,159].

In the context of cancer regulation of inflammatory processes, clinical research on exosomes has brought significant interest due to their involvement in modulating immune responses and inflammation within the tumor microenvironment (NCT01159288 Trial of a Vaccination with Tumor Antigen-loaded Dendritic Cell-derived Exosomes (CSET 1437)). This research highlights the complex interplay between exosomes, inflammation, and cancer progression, offering insights into potential therapeutic strategies for modulating the inflammatory microenvironment in cancer. Studies have investigated how exosomes released by cancer cells can influence immune responses and inflammatory processes, carrying immunomodulatory factors that affect the activity of immune cells [160]. Research focuses on understanding the role of exosomes in promoting inflammation within the tumor microenvironment, carrying pro-inflammatory signals that contribute to a pro-tumorigenic inflammatory milieu [161,162]. Investigations explore how exosomes contribute to the activation or suppression of inflammatory signaling pathways, influencing the overall inflammatory state [161]. Clinical research has also undertaken the potential anti-inflammatory properties of exosomes derived from certain cell types, carrying factors that contribute to resolving inflammation within the tumor microenvironment or modulating immune responses toward an anti-tumorigenic state [163,164]. Ongoing studies explore the possibility of targeting exosomes as a therapeutic strategy to control inflammation in cancer, modulating their release or content to regulate the inflammatory microenvironment within tumors [165].

The EU Clinical Trials Register (https://www.clinicaltrialsregister.eu/ctr-search/search, accessed on 12 January 2024) includes 11 studies, at various levels of advancement, in which exosomes were also taken into account. However, exosomes are not the main subject of interest in these studies. They are isolated and characterized as an additional diagnostic element in the course of effectiveness and safety tests in the treatment of various cancers, mainly prostate (EudraCT Numbers 2018-004458-14, 2015-000270-36, and 2015-001361-27), pancreas (EudraCT Numbers 2015-004860-12 and 2017-003621-15), and kidney (EudraCT Numbers 2011-006009-85 and 2018-001201-93). The information provided indicates a substantial presence of exosome-related clinical trials on https://clinicaltrials.gov/. Out of the 146 registered trials, 66 are interventional, and 80 are observational. Phase 2 trials are the most numerous, with 21 trials, followed by phase 1 trials with 15. Additionally, there are two studies in early phase 1 and phase 3 each. Notably, 31 studies have the status “not applicable,” and a significant portion of 76 studies focuses on examining exosomes for their utility as diagnostic and prognostic markers in cancer. This suggests a diverse and active exploration of exosomes in various phases and contexts within the realm of clinical research, particularly emphasizing their potential diagnostic and prognostic applications in cancer (see Figure 4). Most studies report lung cancer, mainly NSCLC, which accounts for 19% of all described trials. Subsequently, prostate cancer occurs in 14.23% of cases; pancreatic cancer in 8.8%; and breast cancer, primarily with triple-negative characteristics, constitutes 7.5% of cases. Gastric cancer occurs in 5.4%, and head and neck cancer in 4.8% of cases. Other types of cancers collectively make up 40.2% of all cases.

A phase I trial examined the safety and efficacy of exosome-based delivery of paclitaxel (exoPTX) in patients. The findings revealed that exoPTX significantly reduced tumor size with fewer side effects compared to traditional paclitaxel treatment. The overall response rate (ORR) was higher in the exosome group, and patients experienced less severe neuropathy and gastrointestinal toxicity, highlighting the potential of exosome-mediated drug delivery [148]. A pilot study explored exosomes loaded with KRAS G12D siRNA in patients. The treatment effectively silenced the KRAS mutation, resulting in tumor shrinkage in a subset of patients. This approach showed a higher disease control rate compared to conventional chemotherapy, underscoring the potential of exosome-based RNA interference (RNAi) therapies [166]. EV-based therapies have also been investigated for their potential in treating neurological disorders. A clinical trial investigated the use of MSC-derived exosomes in patients with ischemic stroke. The study found that exosome-treated patients had significantly better neurological recovery, as measured by the National Institutes of Health Stroke Scale (NIHSS) and modified Rankin Scale (mRS), compared to those receiving standard post-stroke care. The researchers attributed these improvements to reduced brain inflammation and enhanced neurogenesis [167]. EV-based therapies generally exhibit a superior safety profile compared to traditional treatments. Their natural origin and ability to specifically target disease sites minimize off-target effects and systemic toxicity. For instance, the reduced side effects observed with exosome-mediated paclitaxel delivery underscore this advantage. Exosomes offer enhanced targeted delivery mechanisms, which improve the efficacy of therapeutic agents. This is particularly evident in cancer therapy, where exosomes can deliver chemotherapeutic drugs or genetic material directly to tumor cells. This targeted approach not only improves treatment outcomes but also reduces adverse effects compared to conventional chemotherapy. In regenerative medicine, exosome therapy often outperforms traditional methods by promoting cellular repair and modulating immune responses more effectively. Clinical trials have shown that exosome treatments can achieve better functional recovery and organ repair compared to existing therapies, highlighting their regenerative potential.

EV-based therapies bring promise to healthcare but also raise ethical concerns and risks, particularly concerning patient consent and data privacy, safety, and equitable access. Addressing ethical issues and mitigating potential risks associated with EV-based therapies requires a multifaceted approach involving informed-consent processes, robust data-privacy measures, regulatory compliance, transparent communication, and ethical oversight. By prioritizing patient autonomy, confidentiality, and safety, stakeholders can uphold ethical principles and promote trust in the development and implementation of EV-based therapies.

## 5. Regulatory Affairs of Extracellular Vesicles

In light of business assessments, EVs have great potential for commercialization both toward new diagnostic and therapeutic products. Exosomes’ global market size was estimated to be ca. > USD 250 million in 2022, reaching ca. USD 3.2 billion in 2032 (CARG 2023-2032 29.9%) [168]. Interestingly, the value of the cancer segment for this market is estimated at being over USD 1.2 billion in 2032. Note that EVs-based diagnostics and therapies hold immense market potential and economic impact, driven by advances in biotechnology, personalized medicine, and regenerative medicine. Despite regulatory challenges and market competition, EV-based products are poised to revolutionize healthcare delivery, improve patient outcomes, and stimulate economic growth in the biotechnology sector. Continued investment in research, development, and commercialization efforts is essential to realize the full potential of EV-based technologies and address unmet medical needs effectively.

As it was presented, EVs constitute a promising therapeutic option in many disease areas, including cancer; nevertheless, obtaining the marketing authorization for EV-based medicines in the EU would be a complex process and could be challenging, as currently there are no specific European guidelines for such products. More recently, the U.S. Food and Drug Administration (FDA) announced a work to be done on new guidelines addressing the manufacturing of EV-based products, specifically in regard to mesenchymal stromal cell (MSC)-derived EVs [169]. In a recent announcement, the U.S. FDA also postulated to overcome some limitations related to large-scale EV production and to provide industrial guidelines to meet the demand for EVs to be involved for therapeutic produces [170]. It is a general consensus that the EV-based products fall within the definition of a medicinal product [171]: “Any substance or combination of substances presented for treating or preventing disease in human beings. Any substance or combination of substances which may be administered to human beings with a view to making a medical diagnosis or to restoring, correcting or modifying physiological functions in human beings is likewise considered a medicinal product” (Directive 2001/83/ec) [172]. Also, they are generally considered biological medicinal products, following the definition of the European Medicines Agency (EMA), that this is a medicine whose active substance is made by a living organism [173]. Nevertheless, depending on their manufacturing process, the molecules they carry, and the intended therapeutic use, the EVs may also be classified as biotechnological products or advanced therapy medicinal products (ATMPs).

Currently, a majority of EV-based products being developed or used in clinical trials come from native cells (e.g., mesenchymal stromal stem cells, dendritic cells, and genetically unchanged cells). If the cells carry active substances not intended to introduce any gene modification in the patient’s body, they would most possibly be classified as biological products, even if the methods of priming or stimulation of the naïve cells were applied to produce the EVs. If bioengineering methods are used to obtain the cells producing the EVs (e.g., the cells are immortalized or other genetic modifications are made), but the molecules carried by the vesicles do not fall under the definition of ATMP, the products should most probably be classified as biotechnological products. On the other hand, if the intended therapeutic purpose of the EV-based medicine involves the modification in the recipients’ genome, the product should be classified as a gene therapy medicinal product (GTMP, being part of ATMP; Figure 2) [171,174].

To better understand the differentiation between ATMP and non-ATMP, the scientific recommendations on the classification of such products published by the EMA Committee for Advanced Therapies (CAT) can be looked at. In the list of products published in February 2024, there are, e.g., two medicinal products which contain EVs:

- Human umbilical cord mesenchymal stem cells derived, PEGylated exosomes carrying recombinant hTERT mRNA and proteins;
- Conditioned medium (secretome) from expanded donor bone-marrow mesenchymal stem cells containing cytokines, growth factors, proteins, and extracellular vesicles.

The first one is classified as GTMP, based on the facts that the active substance is a recombinant nucleic acid administered to human beings with a view to regulating a genetic sequence and that its therapeutic effect relates directly to the recombinant nucleic acid sequences it contains. The second product is classified as not ATMP on the basis that it does not contain or consist of recombinant nucleic acids, nor does it contain cells or tissues (Scientific Recommendations on Classification of ATMP, 2024) [175].

In conclusion, in the case of bioengineered EVs, the most appropriate classification would be a biotechnological product or ATMP, depending on its mechanism of action. The issue of classification presented above is important from the point of view of the scope of developmental studies to be performed. It also has implications on the procedural levels, i.e., if the marketing authorization application should be proceeded at the centralized level, by the EMA, or could be proceeded locally. In the case that the product is classified as biologic, the registration can proceed nationally in the Member State selected or through the decentralized procedure in some selected Member States. If it is a biotechnological product or the GTMP, the MA application should be filed to the EMA.

All in all, regardless of the registration procedure selected, the applicant for the MA for the medicinal product in the European Union is required to demonstrate the appropriate quality of the product applied for, its safety for the targeted group of patients, and its efficacy in the proposed indication(s). These are done through the elaborated set of quality and non-clinical studies, and clinical trials, the scope of which is strictly related with the specificity of the medicinal product applied for. This is particularly applicable to such complex products as bioengineered EVs. It should be carefully defined on a case-by-case basis, keeping in mind the provenance of the EVs (“producer cells”), the techniques applied in their manufacturing, the molecules they carry (either in the vesicles or on their surface), and, last but not least, the planned therapeutic action.

As already mentioned, currently there are no specific guidelines issued by the EMA for EV-based medicinal products. Since these products are biologicals, it has been postulated that following the current set of EMA guidelines for biological medicinal products would be sufficient to assure appropriate quality of the EV-based medicinal product (Supplementary Table S1) [173]. There are also guidelines and recommendations prepared by the scientific community involved in the field, e.g., Minimum Information for Studies of Extracellular Vesicles (MISEV) [1], or guidelines prepared by Extracellular Vesicle Translation to Clinical Perspectives—EVOLVE France [171]. The quality documentation should contain such general information as the nomenclature, structure, and general properties of EV-based active substances defined early as active pharmaceutical ingredients [176]. The quality aspects which should be taken into account involve, e.g., establishing a repeatable, validated manufacturing process; the selection of appropriate process controls; and the definition of release criteria, especially for quality, purity and defining chemical and biological impurities (including microbial and viral contamination or TSE agents), potency, and process evaluation and validation. The specifications should address the traits of the origin cells, as well as possible modifications of the EVs and their contents [173,174,177]. The validation of the analytical procedure used for quality control (QC), as well as EV batch analysis, including justification and specification methods used, is a critical step in QS management for EV-based ATMPs. To date, there is no specific information on EV references or EV standards used for specific EV subclasses. For the small- and medium-sized EVs, it would be recommended to also take into account general requirements for nanomedicines (https://www.ema.europa.eu/en/human-regulatory-overview/research-and-development/scientific-guidelines/multidisciplinary-guidelines/multidisciplinary-nanomedicines, accessed on 6 March 2024). Specific care should be taken for stability studies addressing EV-based investigational medicinal products (EV-based IMPs). Note that pristine EVs could behave in different way as compared to bioengineered EVs composed of different added moieties such as genes; proteins; and others, e.g., surface-decorating agents [178]. Therefore, the storage, description, and composition of EV-based IMPs matter, including the pharmaceutical and biotechnological development and the description of their manufacture based on GMP standards, batch formulae, control of excipients—especially of human or animal origin—if any are used in the formulation, and finally activities related to facilities and equipment used in the manufacturing process and applied contained closure system. It should be emphasized that the validation of the analytical procedure for all methods and tests used in a quality-control system will be required in laboratory-based standards and certifications. This should be especially considered for all novel methods and assays developed for EV characterization [179].

**Figure 5:**
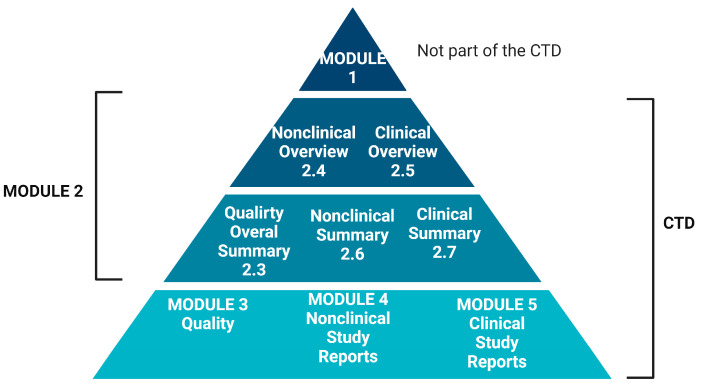
The CTD triangle. Abbreviations: CTD—Common Technical Document.

In general, the application of the risk-based approach, as per the “Guideline on quality, non-clinical and clinical requirements for investigational advanced therapy medicinal products in clinical trials—Scientific guideline” (EMA/CAT/852602/2018) [180] and “ICH guideline Q9 on quality risk management” (EMA/CHMP/ICH/24235/2006) [181], is recommended when working on the manufacturing process and controls. To date, nonclinical documentation for EV-based medicinal products, as required in the Common Technical Document (CTD) [182,183], should provide information on non-clinical models, the general outline of the non-clinical development, and the timing of non-clinical studies (Figure 5).

It is obvious that the non-clinical development pathway for EV-based medicinal products, especially EV-based ATMPs, is significantly different for other medicinal products based on simple chemical agents. In general, the non-clinical data should provide information on the efficacy and safety of the biological active dose; therefore, in vivo animal studies should be carefully planned to ensure the generation of robust data, while considering the 3R (reduction, replacement, and refinement) rules. The use of 3D cultures, especially advanced organoids and lab-on-chip systems, should be involved in such studies [184]. In preclinical pharmacology studies, the dose level for proof of concept should allow for the estimation of the biologically effective dose (BED) to help establish a clinical effecting dose (CED). It is also important to establish the BED with an acceptably safety level of the EV-based product. Therefore, toxicology studies supported with pharmacokinetic profiling should be applied as minimum non-clinical data requirements before first-in-human studies. In non-clinical safety studies, such as systemic toxicity, a primary focus before clinical setups, the need for additional toxicity studies, such as genotoxicity, tumorigenicity, reproductive, and developmental toxicity, as well as immunotoxicity studies, should be determined on a case-by-case basis, considering the risk of the particular class of EV-based products in clinical scenarios. Therefore, general toxicity studies should provide information for the estimation of the safe starting dose and dosing regimen and identify relevant safety concerns. Standard genotoxicity assays are generally not appropriated for EV-based ATMPs; however, the specific toxicological concerns, such as genome modifications due to insertional mutagenesis, should be taken into considerations. It seems that standard lifetime rodent carcinogenicity studies will not be required; however, the tumorigenic and teratogenic potential should be tested using in vitro models for neoplasm signals, oncogenic activation, or the cell proliferation index. It is generally accepted that immune responses of the innate and adaptive systems should be examined in different EV-treated models. In general, repeated-toxicity studies will support multiple-dose regiments in human subjects. This will allow us to collect the data for long-term adverse effects, if any.

Importantly, EMA strongly recommends discussing the development plan of all biological medicinal products with the agency through the procedure of Scientific Advice. Having in mind the complexity of the EV-based therapeutic products and the room for interpretation of the currently available guidelines, it would be reasonable to enter into discussion with the authorities early in the development.

The range of required non-clinical studies depends on the intended purpose of the therapeutic EV and its classification. In general, the recommendations of “Strategies to identify and mitigate risks for first-in-human and early clinical trials with investigational medicinal products—Scientific guideline” (EMEA/CHMP/SWP/28367/07) [185] should be followed; however, in case of products to be used, e.g., in cancer, the “ICH S9 guideline on non-clinical evaluation for anticancer pharmaceuticals” (EMA/CHMP/ICH/646107/2008) [186] would be applicable. In the case of EV-based products, which will be classified as biotechnological, the “ICH S6 (R1) guideline on preclinical safety evaluation of biotechnology-derived pharmaceuticals” (CPMP/ICH/302/95) [187] should be applied. If the developed product falls under the definition of the GTMP, the applicable guidelines on quality, non-clinical and clinical requirements for advanced-therapy medicinal products should also be taken into account (Supplementary Table S1). Importantly, the product tested in non-clinical studies, especially the toxicology studies, should be representative of that, which is intended to be used in clinical trials. If possible, it is recommended to use the formulation of the product, which is similar to that intended for human subjects. As an exception rather than the rule, in some early pharmacokinetic (PK) or pharmacodynamic (PD) studies, a more preliminary formulation of the product can be used. The non-clinical studies should bring convincing proof that the developed medicinal product is safe to be administered to human subjects (safety pharmacology and toxicology studies) and that it likely to exert the intended therapeutic effect (PK and PD studies). Currently, a majority of EV-based products are of allogenic origin; therefore, the assessment of the immunogenic potential of the products is of particular importance. The pivotal safety studies (safety pharmacology, toxicology, and immunogenicity) are required to be performed in the Good Laboratory Practice (GLP) certified facility(-ies). The extent of the PD studies to be performed depends on the planned therapeutic indication. If possible, the demonstration of the proof of concept with the use of a suitable animal model of the disease should be executed. These studies do not need to be performed in a GLP-certified facility; however, the compliance with GLP recommendations would be highly beneficial.

In terms of clinical development, general principles that apply to any investigational medical product established in the “Regulation (EU) No 536/2014 of the European Parliament and of the Council of 16 April 2014 on clinical trials on medicinal products for human use, and repealing Directive 2001/20/EC Text with EEA relevance” (Regulation 536/2014) [188] should be considered. The scope of the clinical development program would depend on the planned therapeutic use of the EV-based product. Given the complex nature of the products, the scientific community recommends to follow the guidelines for investigational advanced therapy medicinal products, as per the “Guideline on quality, non-clinical and clinical requirements for investigational advanced therapy medicinal products in clinical trials—Scientific guideline” (EMA/CAT/852602/2018) [180], especially at the early stages of the development. In later stages (phase II/III), the target disease-specific guidelines should be followed, which can be found on the EMA website. Generally, the aim of the clinical program would be to determine the safety and efficacy of the developed product, the dosing regimen recommended for therapy, the spectrum of possible adverse events, and the limitations of the products use (e.g., in pregnancy, while breast-feeding). For exploratory clinical trials, especially for the First-in-Human (FIH) studies, the primary objective should be focused on safety and the tolerability effects. Therefore, the design of the exploratory clinical studies of EV-based ATMPs should involve extended or permanent adverse effects, e.g., immunosuppressive or immunostimulatory effects, integration into the genome, or even malignant transformation. Since the EVs are derived from human cells/tissues and are mostly composed of naturally occurring substances, their safety is considered rather high. Nevertheless, as it was mentioned before that the potential for immunogenicity should definitely be examined (in case of allogenic provenance of EVs). Of note, given the biologic source of the EVs, there might be limitations of current techniques for their tracking and imaging within cells and tissues. Therefore, the determination of their pharmacokinetics and biodistribution may be challenging. For data purposes, the labeling of EVs using different dyes should be used to study cellular uptakes (in vitro) or in vivo biodistribution using specific dyes and modalities [180].

The legal requirements and principle regulatory guidelines to be taken into account during the development; the non-clinical studies and clinical trials of EV-based medicinal products, as per the scientific community recommendations [171,173,174]; and the authors’ expertise are described in Supplementary Table S1.

The applicable guidelines may differ depending on the specific characteristics of the developed product, and the scope should be adjusted on a case-by-case basis. Also, the list is not exhaustive; as each product has its individual particulars, other specific guidelines may also apply. As presented in the text, for bioengineered EVs, the guidelines for biotechnological products and, in some cases, ATMP are generally applicable. The ATMP guidelines, which may be followed but are not mandatory for non-ATMP EV-based products are marked with an asterisk (*). Nevertheless, it is generally recommended by the scientific community to take into account the recommendations for ATMPs in the development of EVs, even if the product is not strictly classified as an ATMP. The scope of clinical guidelines depends on the planned indication of the product. In Supplementary Table S1, guidelines addressing general considerations for clinical trials are presented, plus the guidelines related to potential cancer therapy. The design of clinical studies for EV-based medicinal products, including EV-based ATMPs, deserves specific considerations. Because the data from non-clinical, pharmaco-dynamic, pharmacokinetic, and toxicity studies may be limited to the specific human situation and diseases, this may hamper, among other things, the prediction of a safety starting dose for FIH trials (EMEA/CHMP/SWP/28367/07 Rev. 1). Therefore, the risk assessment in pre- and post-marketing surveillance should take into consideration for numerous factors collected in non-clinical and clinical phases. It seems reasonable to conclude that the pharmacovigilance program is optional for all new EV-based medical products released on global markets, and this could be encompassed by the Marketing Authorization Holder in a dossier presenting the strategy for post-marketing activities [189,190]. This strategy requires us to develop a Pharmacovigilance System Mater File (FSMF) and the Risk Management Plan (RMP). The legal basis for regulatory approval in the European Union to address numerous regulations is presented in Supplementary Table S1. The EV-based medicinal products provide new possibilities for restoring, correcting, or modifying cellular and molecular functions, or making some novel diagnosis. At the same time, because of their novelty, complexity, and specificity, they may produce new risks to patients. The risks for EVs identified in non-clinical and clinical studies should be encompassed within risk management plans, connecting both risk communication and risk-mitigation systems [189,191]. This may include risks to patients in relation to the quality, safety, storage, and distribution of the EV product; risks to patients in relation to specific diseases and mode of treatments; and risks to patients in relation to the environment, especially when EV-based GMO products are developed. Therefore, more regulatory standards are still required to identify such risks and provide a global regulatory platform for the effective mitigation of their adverse effects to patients. In other words, the detection of the risk due to EV-based medicinal products should start early in non-clinical and quality studies before FIH, and novel strategies and technologies, such as safety-by-design methods, lab-on-a-chip, and even artificial intelligence, should be applied in future research and development programs following some regulatory guidelines.

## 6. Future Perspectives

Exosomes, with their unique properties and potential applications, have garnered interest not only in biomedicine but also in related fields, such as bioengineering, materials science, and pharmacology. Bioengineers explore methods to modify exosomes for targeted drug delivery, improved stability, and controlled release. Techniques like surface functionalization, lipid modification, and encapsulation enable the customization of exosomes with specific properties for biomedical uses. Exosomes inspire the design of biomimetic nanoparticles, mimicking their structure and function for drug delivery and tissue engineering. Synthetic vesicles and liposomes developed by bioengineers resemble exosomes’ membrane composition and surface markers, enhancing biocompatibility and targeting. Materials scientists design and optimize biomaterial-based platforms for the efficient isolation and purification of exosomes from complex biological samples. Nanomaterials, microfluidic devices, and functionalized surfaces offer precise control over exosome capture and separation for downstream applications. Researchers study interactions between exosomes and various materials, like nanoparticles, polymers, and scaffolds, to understand their effect on exosome stability, cargo delivery, and therapeutic efficacy. Material properties such as surface chemistry, topography, and mechanical properties influence exosome binding, uptake, and intracellular trafficking in recipient cells. Pharmacologists explore exosomes as natural drug delivery vehicles for therapeutic compounds like small molecules, nucleic acids, and biologics. Exosome-mediated drug delivery offers benefits such as enhanced bioavailability, prolonged circulation, and targeted delivery to specific tissues or cells. Pharmacologists investigate exosome-mediated intercellular communication’s role in modulating physiological processes, disease progression, and drug response. Exosomes act as carriers of signaling molecules, cytokines, and growth factors, influencing cellular signaling pathways and pharmacological responses in recipient cells.

Extracellular vesicles as membrane-bound particles secreted by cells that hold significant promise in shaping the future of diagnostics and ATMPs. As our comprehension of EVs deepens, their potential to revolutionize these fields becomes increasingly evident. In the field of diagnostics, EVs serve as carriers of a diverse molecular cargo reflective of their cell of origin, enclosing proteins, nucleic acids, and lipids. This inherent characteristic renders them invaluable biomarkers for a spectrum of diseases. Emerging diagnostic methodologies involve isolating EVs from bodily fluids like blood or urine and scrutinizing their cargo for disease-specific signatures. Diseases, ranging from cancer to neurodegenerative disorders and infectious diseases, stand to benefit from such EV-based diagnostic approaches. By facilitating earlier disease detection, enabling personalized medicine strategies, and facilitating real-time treatment response monitoring, EV-based diagnostics promise to usher in a new era of healthcare (Figure 2).

Simultaneously, EVs hold immense potential in the domain of ATMPs. Compared to traditional gene-based or cell-based therapies, the EV medicinal products present a compelling alternative owing to their diminished immunogenicity, smaller size, and remarkable ability to traverse biological barriers, including the formidable blood–brain barrier. Furthermore, EVs can be tailored to carry therapeutic payloads such as drugs, nucleic acids, or proteins with precision to target cells. Future ATMPs may render EVs useful as versatile drug delivery vehicles to address innumerable conditions, including cancer, neurological disorders, and inflammatory diseases. By offering safer, more efficacious treatment options with reduced side effects compared with conventional therapies, EV-based therapies hold the promise of significantly enhancing patient outcomes.

Despite these prospects, the full realization of EVs’ clinical potential necessitates concerted efforts in further research and development. More studies should be performed on standardization methods used for upstream and downstream processes in EV production based on large-scale manufacturing. Quality, safety, and efficacy programs need a global regulatory approval, especially for those EVs used as advanced therapy medicinal products in rare diseases, cancers, and regenerative medicines. For preclinical and clinical safety studies, both toxicology and risk management should be defined for EV-based medicines. Challenges representative of scalability, standardization of methodologies, and regulatory approval frameworks must be diligently addressed to facilitate widespread adoption in clinical settings. Nonetheless, the paths of EVs in diagnostics and ATMPs promise transformative changes in healthcare, characterized by early disease interception, tailored therapeutic interventions, and overall improved patient well-being. Future research directions and emerging trends in extracellular vesicle (EV) research offer promising avenues for expanding our understanding of EV biology and harnessing EVs’ therapeutic and diagnostic potential. Precision medicine holds promise in exploring novel EV biomarkers for early disease detection and treatment-response prediction, while personalized EV-based therapies tailored to individual patient profiles could revolutionize treatment strategies. Advanced technologies, such as engineering EVs with customizable cargo and targeting ligands and improving imaging and detection techniques for real-time visualization and tracking of EVs, offer exciting opportunities for innovation. Bioproduction and manufacturing optimization are essential for scaling up EV production for clinical-grade applications, while standardized protocols and quality-control measures ensure product consistency and safety. Clinical translation and commercialization efforts, supported by collaboration between academia, industry, and regulatory bodies, are critical for bringing EV-based diagnostics and therapies to patients. Effective collaboration fosters a synergistic ecosystem that accelerates innovation and improves patient outcomes in diverse fields of healthcare.

## 7. Conclusions

Extracellular vesicles emerge as promising candidates in shaping the future of diagnostics and ATMPs. Clinical research on EVs represents a multidimensional approach with profound implications for cancer diagnostics, gene therapy, drug delivery, neurology applications, and the regulation of inflammatory processes. The cumulative knowledge from these studies holds the potential to initiate a new era of diagnostic and therapeutic strategies, offering innovative solutions in the treatment of diverse conditions associated with cancer. Their ability to serve as carriers of diverse molecular cargo reflective of their cell of origin positions them as invaluable biomarkers for a wide array of diseases, offering opportunities for earlier disease detection and personalized medicine approaches. Moreover, EVs exhibit significant potential in the area of ATMPs, presenting an alternative to traditional gene-based and cell-based therapies due to their advantageous properties, such as reduced immunogenicity and the ability to cross biological barriers. Despite these promising prospects, addressing challenges related to scalability, standardization, and regulatory approval is imperative to realize the full clinical potential of EVs. Nevertheless, the path of EVs in diagnostics and ATMPs suggests transformative changes in healthcare, including early disease interception, tailored therapeutic interventions, and improved patient outcomes.

## Figures and Tables

**Table 1:** Simplified EVs’ manufacturing process.

	Objective	Process 1	Process 2	Quality Control
Cell culturing	Grow the cells that will produce the EVs	Select a cell type	Culture these cells in flasks or bioreactors under controlled conditions	Authentication, purity assessment
EVs production	Stimulate the cells to produce and release EVs	Once cells reach a certain density, induce exosome production through specific stimuli	Allow cells to secrete EVs into the culture medium over a period of time	Authentication, purity assessment
Harvesting EVs	Collect the EV-rich culture medium	Collect the culture medium containing the EVs	Remove the cells from the medium through centrifugation or filtration	Authentication, purity assessment
EVs isolation	Purify the EVs	Perform differential centrifugation	Use ultracentrifugation or Size-Exclusion Chromatography to isolate EVs based on size and density	Method validation, contaminant removal
EVs characterization	Ensure the EVs meet quality and functional standards	Analyze the size, concentration, and surface markers of the EVs using NTA and flow cytometry	Confirm the presence of specific proteins or RNA	Size distribution, quantification, cargo profiling
EVs packaging and storage	Prepare the EVs for storage and distribution	Sterile filter the EVs preparation to ensure it is free from contaminants	Package the EVs in sterile vials and store at −80 °C or in liquid nitrogen	Biological activity, safety evaluation, storage conditions

Abbreviations: EVs—extracellular vesicles; NTA—Nanoparticle Tracking Analysis; RNA—ribonucleic acid.

**Table 2:** Comparison of different EV isolation techniques, taking into account the advantages and disadvantages of each method.

Method	Process	Pros	Cons
Differential centrifugation	Sequentially spins samples at increasing speeds to remove cells, debris, and larger vesicles, eventually pelleting EVs	- Widely used - Large volumes - No special equipment	- Time-consuming - Limited specificity; co-isolate other particles - Potential aggregation
Ultracentrifugation	Uses very high-speed centrifugation to pellet EVs based on their density	- Highly effective for small EVs - High purity with density gradient	- Expensive - Potential EV damage - Time-intensive
SEC	Separates EVs based on size as they pass through a column with porous beads	- Gentle on EVs - High purity - Reproducible and scalable	- Limited sample volume - Specialized chromatography columns - Time-consuming
Polymer-based precipitation	Uses polymers (e.g., polyethylene glycol) to precipitate EVs from solution	- Simple and quick - No special equipment - Suitable for a wide range of sample volumes	- Co-precipitates contaminants - Lower purity - Needs further purification
Ultrafiltration	Uses membrane filters to separate EVs based on size	- Rapid - Scalable - No centrifugation. - Suitable for processing large volumes	- Membrane clogging - Potential EVs damage - Multiple steps for purity
Immunoaffinity capture	Uses antibodies specific to EV surface markers to capture EVs from a mixture	- High specificity - Can isolate subpopulations - High purity	- Expensive - Small volumes - Requires knowledge of markers
Microfluidics	Utilizes microfluidic devices to isolate EVs based on size, charge, or other properties	- High precision - Rapid - Integrates with analytics	- Specialized equipment - Limited scalability - Complex development

Abbreviations: EVs—extracellular vesicles; SEC—Size-Exclusion Chromatography.

## Data Availability

Not applicable.

## Supplementary Materials

The following supporting information can be downloaded at https://www.mdpi.com/article/10.3390/ijms25126533/s1.
