# Supplementary material for: Extracellular Vesicles as Next-Generation Diagnostics and Advanced Therapy Medicinal Products
Source: Int J Mol Sci. 2024 Jun 13;25(12):6533. doi: 10.3390/ijms25126533 (PMC11204223; doi:10.3390/ijms25126533)
Supplement: Supplementary file 1 [file ijms-25-06533-s001.zip › ijms-3008328-supplementary.pdf]

International Journal of *Molecular Sciences*

MDPI

Supplementary Materials

Extracellular vesicles as next generation diagnostics and advanced therapy medicinal products

Agnieszka Stawarska [1*], Magdalena Bamburowicz-Klimkowska [1], Elise Runden-Pran [2], Maria Dusinska [2], Mihaela Roxana Cimpan [3], Ivan Rios-Mondragon[3] , and Ireneusz P. Grudzinski [1]

[1] Department of Toxicology and Food Science, Faculty of Pharmacy, Medical University of Warsaw, Banacha Str. 1, PL-02-097 Warsaw, Poland

[2] Health Effects Laboratory, Department of Environmental Chemistry, Norwegian Institute for Air Research, 2007 Kjeller, Norway

[3] Biomaterials - Department of Clinical Dentistry, Faculty of Medicine, University of Bergen, Årstadveien Str. 19, Bergen 5009, Norway

[*] Correspondence to: agnieszka.stawarska@wum.edu.pl

International Journal of *Molecular Sciences*

MDPI

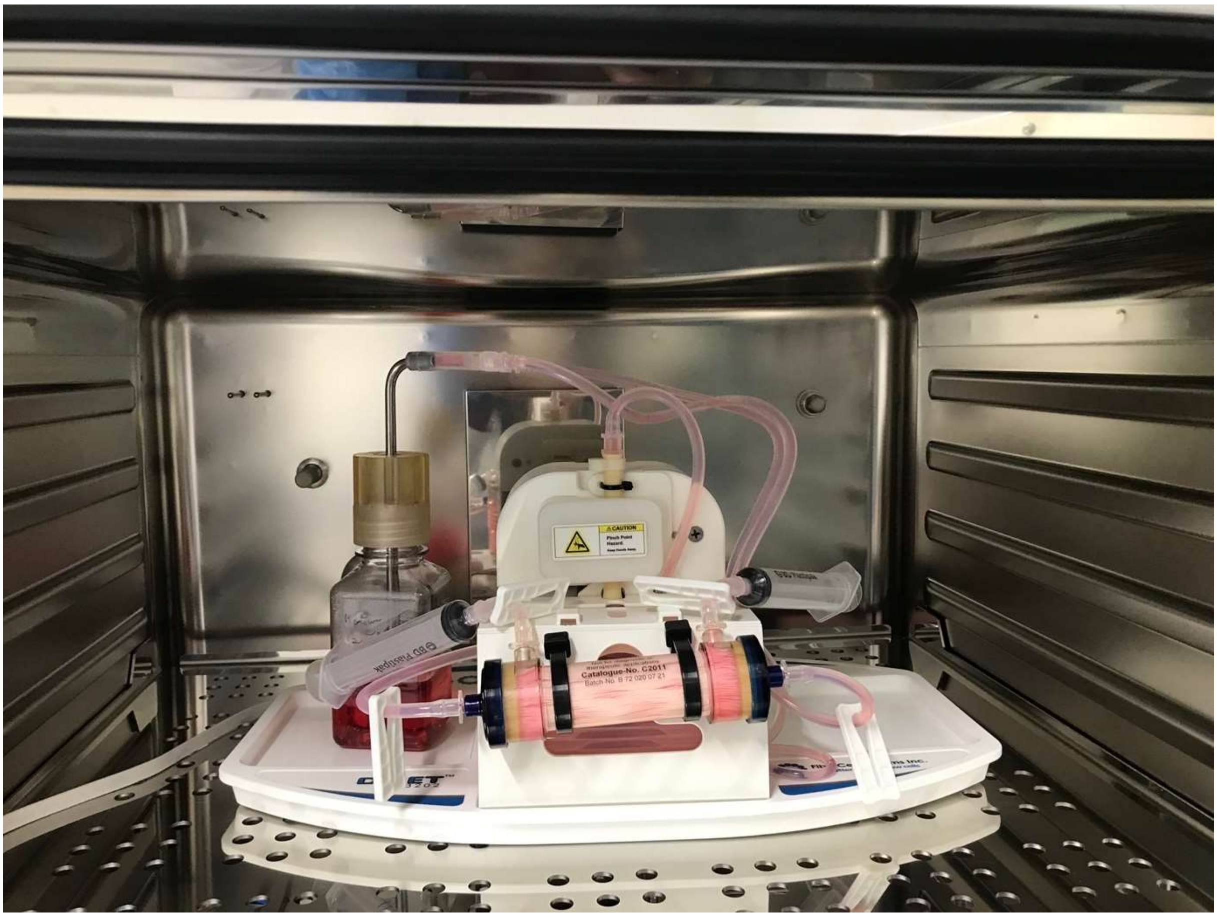

Figure S1. A photo of the hollow fiber bioreactor (HFB). The media reservoir, peristaltic pump, and an extralong silicone tube, acting as an oxygenator, are connected to the intracellular space of the HFB cartridge. The HFB system is operated at the Cell and Tissue Laboratory of the Department of Toxicology and Food Science, Faculty of Pharmacy, Medical University of Warsaw, Warsaw, Poland.

International Journal of *Molecular Sciences*

MDPI

Table S1. The legal requirements and principle regulatory guidelines to be taken into consideration during the pharmaceutical development, non-clinical studies and clinical trials of EV-based medicinal products.

Reference number	Guideline title	Effective date
**Pharmaceutical development / quality**		
ICH Q2(R2)	Validation of analytical procedures	1 November 2023
ICH Q5A(R2)<br>EMA/CHMP/ICH/804363/2022	Guideline on viral safety evaluation of biotechnology products derived from cell lines of human or animal origin	14 June 2024
ICH Q5C	Quality of biotechnological products: stability testing of biotechnological/ biological products	30 November 1995
ICH Q5D	Derivation and characterisation of cell substrates used for production of biotechnological/ biological products	16 July 1997
ICH Q5E	Comparability of biotechnological/ biological products subject to changes in their manufacturing process	18 November 2004
ICH Q6B<br>CPMP/ICH/365/96	Specifications: test procedures and acceptance criteria for biotechnological/ biological products	1 September 1999
ICH Q7	Good manufacturing practice guide for active pharmaceutical ingredients	10 November 2000
ICH Q9(R1)	Quality risk management	18 January 2023
EMA/CHMP/BWP/706271/2010	Plasma-derived medicinal products	1 February 2012
EMEA/CHMP/410869/2006 (*)	Human cell-based medicinal products	1 September 2008
EMA/CAT/571134/2009 (*)	Stem cell-based medicinal products	14 January 2011
CPMP/BWP/1143/00	Position statement on the use of tumorigenic cells of human origin for the production of biological and biotechnological medicinal products	February 2001
EMA/CHMP/BWP/187338/2014	Guideline on process validation for the manufacture of biotechnology-derived active substances and data to be provided in the regulatory submission	1 November 2016

3ab1aen	Production and quality control of medicinal products derived by recombinant DNA technology	1 July 1995
EMA/CHMP/BWP/729106/2011	Guideline on the use of starting materials and intermediates collected from different sources in the manufacturing of non-recombinant biological medicinal products	1 December 2013
3ab6a (*)	Gene therapy product quality aspects in the production of vectors and genetically modified somatic cells	1 July 1995
EMA/CAT/499821/2019 (*)	Questions and answers on comparability considerations for advanced therapy medicinal products (ATMP)	Adopted on 6 December 2019
EMEA/410/01 Rev. 3	Note for guidance on minimising the risk of transmitting animal spongiform encephalopathy agents via human and veterinary medicinal products	1 July 2011
GMP annex 13	Manufacture of Investigational Medicinal Products	31 July 2010
GMP annex 13	Detailed Commission guideline of 8 December 2017 on the good manufacturing practice for investigational medicinal products pursuant to the second paragraph of the Article 63(1) of Regulation (EU) No 536/2014	The Regulation 536/2014 is fully in force since 31 January 2023
EMA/CHMP/BWP/534898/2008 Rev. 2	Requirements for quality documentation concerning biological investigational medicinal products in clinical trials	31 January 2022
EMEA/CHMP/BWP/398498/05	Virus safety evaluation of biotechnological investigational medicinal products	1 February 2009
EMA/CAT/852602/2018 (*)	Guideline on quality, non-clinical and clinical requirements for investigational advanced therapy medicinal products in clinical trials	Draft adopted in January 2019
ICH M4 (Quality dossier)	Common Technical Document	November 2000
**Non-clinical development**		
ICH S6 (R1)	Preclinical safety evaluation of biotechnology-derived pharmaceuticals	Parent Guideline dated 16 July 1997<br>Addendum incorporated in June 2011

EMEA/CHMP/SWP/28367/07 Rev. 1	Guideline on strategies to identify and mitigate risks for first-in-human and early clinical trials with investigational medicinal products	01 February 2018
ICH S9	Non-clinical evaluation for anticancer pharmaceuticals	29 October 2009
EMEA/CHMP/GTWP/125459/2006 (*)	Non-clinical studies required before first clinical use of gene therapy medicinal products	November 2008
ICH M3 (R2)	Guidance on non-clinical safety studies for the conduct of human clinical trials and marketing authorization for pharmaceuticals	11 June 2009
ICH S2 (R1)	Guidance on genotoxicity testing and data interpretation for pharmaceuticals intended for human use	9 November 2011
CPMP/SWP/1042/99 Rev 1 Correction	Guideline on repeated dose toxicity	01 September 2010
ICH S3A	Note for guidance on toxicokinetics: the assessment of systemic exposure in toxicity studies	27 October 1994
ICH S4<br>CPMP/ICH/300/95	Duration of chronic toxicity testing in animals (rodent and non-rodent toxicity testing)	1 May 1999
ICH S3B	Pharmacokinetics: Guidance for repeated dose tissue distribution studies	June 1995
ICH S7A	Safety pharmacology studies for human pharmaceuticals	8 November 2000
ICH S7B	The non-clinical evaluation of the potential for delayed ventricular repolarization (QT interval prolongation) by human pharmaceuticals	12 May 2005
ICH S12 (*)	Non-clinical biodistribution considerations for gene therapy products	14 March 2023
ICH M10	Bioanalytical method validation and study sample analysis	24 May 2022
EMA/CHMP/SWP/169215/2005	Need for non-clinical testing in juvenile animals on human pharmaceuticals for paediatric indications	01 August 2008
ICH M4 (Non-clinical dossier)	Common Technical Document	November 2000
**Clinical development** *(the scope of guidelines depends vastly on the planned indication)*		
Regulation (EU) No 536/2014	Regulation (EU) No 536/2014 of the European Parliament and of the Council of 16 April 2014 on clinical trials on medicinal products for	Adopted on 16 June 2014

International Journal of
Molecular Sciences

	human use, and repealing Directive 2001/20/EC	Fully in force since 31 January 2023
ICH E6(R2)	Integrated addendum to ICH E6(R1): guideline for good clinical practice	9 November 2016
ICH E8(R1)	General considerations for clinical studies	6 October 2021
ICH E9	Statistical principles for clinical trials	5 February 1998
ICH E1<br>CPMP/ICH/375/95	Population exposure: The extent of population exposure to assess clinical safety	June 1995
ICH E10	Choice of control group and related issues in clinical trials	20 July 2000
E2A-E2F	Pharmacovigilance	1994 - 2012
ICH E4	Dose-response information to support drug registration	10 March 1994
ICH E3	Structure and content of clinical study reports	30 November 1995
ICH E16	Biomarkers related to drug or biotechnology product development: context, structure and format of qualification submissions	20 August 2010
E18	Guideline on genomic sampling and management of genomic data	3 August 2017
EMEA/CHMP/EWP/192217/2009 Rev.1 Corr.2**	Bioanalytical method validation	1 February 2012
3CC3a	Pharmacokinetic studies in man	1 October 2015
EMA/CHMP/205/95 Rev.6	Guideline on the clinical evaluation of anticancer medicinal products	Adopted on 18 November 2023
CHMP/EWP/83561/2005	Guideline on clinical trials in small populations	1 February 2007
EMEA/149995/2008 (*)	Guideline on safety and efficacy follow-up and risk management of advanced therapy medicinal products	1 January 2009
ICH M4 (Clinical dossier)	Common Technical Document	November 2000
**General requirements for ATMP, which may be adopted for EVs**		
Regulation EC/1394/2007	Regulation on advanced therapy medicinal products.	30 December 2007

International Journal of
Molecular Sciences

MDPI

EMA/CAT/600280/2010 Rev. 1	Reflection paper on classification of advanced therapy medicinal products	Adopted on 22 May 2015
EMA/CAT/CPWP/686637/2011	Guideline on the risk-based approach according to annex I, part IV of Directive 2001/83/EC applied to Advanced therapy medicinal products.	12 February 2013
EMA/CAT/80183/2014	Guideline on the quality, non-clinical and clinical aspects of gene therapy medicinal products	Draft adopted in March 2018
